# Distinct Bacterial Microbiomes in Sexual and Asexual *Potamopyrgus antipodarum*, a New Zealand Freshwater Snail

**DOI:** 10.1371/journal.pone.0161050

**Published:** 2016-08-26

**Authors:** Cristina Takacs-Vesbach, Kayla King, David Van Horn, Katelyn Larkin, Maurine Neiman

**Affiliations:** 1 Department of Biology, University of New Mexico, Albuquerque, New Mexico, United States of America; 2 Department of Zoology, University of Oxford, Oxford, United Kingdom; 3 Department of Biology, University of Iowa, Iowa City, Iowa, United States of America; Argonne National Laboratory, UNITED STATES

## Abstract

Different reproductive strategies and the transition to asexuality can be associated with microbial symbionts. Whether such a link exists within mollusks has never been evaluated. We took the first steps towards addressing this possibility by performing pyrosequencing of bacterial 16S rRNA genes associated with *Potamopyrgus antipodarum*, a New Zealand freshwater snail. A diverse set of 60 tissue collections from *P*. *antipodarum* that were genetically and geographically distinct and either obligately sexual or asexual were included, which allowed us to evaluate whether reproductive mode was associated with a particular bacterial community. 2624 unique operational taxonomic units (OTU, 97% DNA similarity) were detected, which were distributed across ~30 phyla. While alpha diversity metrics varied little among individual samples, significant differences in bacterial community composition and structure were detected between sexual and asexual snails, as well as among snails from different lakes and genetic backgrounds. The mean dissimilarity of the bacterial communities between the sexual and asexual *P*. *antipodarum* was 90%, largely driven by the presence of Rickettsiales in sexual snails and *Rhodobacter* in asexual snails. Our study suggests that there might be a link between reproductive mode and the bacterial microbiome of *P*. *antipodarum*, though a causal connection requires additional study.

## Introduction

The production of offspring is one of the most important determinants of organismal fitness. Despite the tight links between reproduction and fitness, the mechanisms of offspring production are characterized by remarkable inter- and intraspecific variation. It is increasingly evident that different reproductive strategies, such as host mating behaviour [1–3] and the production of viable offspring [4], can be associated with the composition of an organism’s microbiome. The transition from sexual to asexual reproduction can be driven by infection of sexual females with endosymbiotic bacteria [5–10], and links between asexuality and bacterial endosymbionts have been established in many arthropod taxa (reviewed in [5–7,9–11]) and in nematodes [12] (and reviewed in [5]). Together, these studies demonstrate that the microbiome is an integral component of host reproductive biology for at least some animals, though connections between microbial communities and reproductive mode in other animal taxa remain largely unexplored.

Here, we compare the microbiome community structure between obligately sexual and obligately asexual *Potamopyrgus antipodarum*, a New Zealand freshwater snail, to determine if reproductive mode variation in this system might be associated with a particular bacterial community. *Potamopyrgus antipodarum* is a powerful system for evaluating potential connections between reproductive mode and microbiota because natural populations of this snail vary widely in the relative frequency of obligately sexual individuals (male and female) and obligately asexual individuals (nearly always female; males are produced by asexual females at a rate of 1–2% of all offspring [13–15]). Like many mixed sexual/asexual animal taxa (reviewed in [16]), asexual *P*. *antipodarum* are typically polyploid (triploid and tetraploid [17]), while sexuals are diploid [18]. The rare occurrence of diploid asexual *P*. *antipodarum* suggests that polyploidy is a consequence rather than a cause of asexuality in *P*. *antipodarum*, with polyploidy arising via fertilization of the unreduced asexually produced eggs produced by asexual females [17]. Phylogeographic [17,19] and population genetic [20,21] data indicate that asexual lineages of *P*. *antipodarum* have evolved multiple times from sexual populations of *P*. *antipodarum*, thereby providing a replicated system to evaluate links between reproductive mode and microbiome composition within a single species.

## Materials and Methods

### Host Tissue Collection, Identification, and Preparation

We used barcoded amplicon pyrosequencing of 16S rRNA genes to characterize the bacterial communities associated with a diverse array of laboratory-cultured and field-collected *P*. *antipodarum*. Laboratory-cultured snails had been housed at the University of Iowa for at least 2–3 generations; the eight laboratory lineages that we used (two sexual diploid, “2x”; three asexual triploid, “3x”; three asexual tetraploid, “4x”) were each descended from the offspring produced by a single field-collected female that originated from the littoral zone (maximum depth 1m) of the South Island New Zealand lakes Alexandrina, Gunn, Kaniere, Poerua, Rotoiti, and Taylor (Table 1). Field-collected *P*. *antipodarum* samples in this study were collected in January 2011 from the South Island New Zealand lakes Ianthe, Kaniere, and Sarah (maximum depth 1m). We made an effort to select laboratory lineages and field collections that represented genetically and geographically distinct source populations and that allowed us to compare separately derived asexual lineages [17,21]. We hereafter refer to each distinct laboratory lineage and field collection as a “population”.

**Table 1:** Characteristics of the 11 populations and sample types used in this study.

Lake^a^^,^^b^	Latitude	Longitude	Sample Type	Reproductive Mode	Ploidy	# ♀ head^b^	# ♀ body^b^	#♂ head^b^	#♂ body^b^	Juvenile^c^	Total
Alexandrina	43.9500°S	170.4500°E	Laboratory lineage	Sex	2x	1	1	1	1	0	4
Gunn	44.8833°S	168.0833°E	Laboratory lineage	Asex	4x	1	1	1	1	0	4
Gunn	44.8833°S	168.0833°E	Laboratory lineage	Asex	4x	1	1	1	1	0	4
Ianthe	43.0500°S	170.6167°E	Field collection	Sex	2x	4	4	4	4	0	16
Kaniere	42.8333°S	171.1500°E	Field collection	Sex	2x	1	1	1	1	0	4
Kaniere	42.8333°S	171.1500°E	Laboratory lineage	Sex	2x	1	1	1	1	0	4
Poerua	42.7000°S	171.5000°E	Laboratory lineage	Asex	3x	1	1	1	1	0	4
Poerua	42.7000°S	171.5000°E	Laboratory lineage	Asex	3x	1	1	1	1	0	4
Rotoiti	38.0390°S	176.4277°E	Laboratory lineage	Asex	4x	1	1	1	1	0	4
Sarah	43.0500°S	171.7667°E	Field collection	Asex	3x	4	0	0	0	4	8
Taylor	42.4500°S	172.1600°E	Laboratory lineage	Asex	3x	1	1	1	1	0	4
											**60**

^a^Represents lake of origin for founding female of laboratory lineage and lake of collection for field-collected samples.

^b^Each sample except those from Ianthe and Sarah represents tissue from 3 pooled individual snails; head and body tissue subsamples were analyzed separately except for juveniles, which are so small that we combined head and body tissue for DNA extraction. Ianthe and Sarah snails were analyzed individually.

^c^Juveniles are too young to sex. These juveniles were triploid and as such were presumed to be female. One head half and the body tissue were combined for each juvenile to ensure that we had enough tissue for DNA extraction.

We arbitrarily selected three males and three females (i.e., six snails per lineage) from each of the eight laboratory lineages (two sexual and six asexual, including two distinct asexual lineages from Gunn and two distinct asexual lineages from Poerua) and the Kaniere (sexual) field-collected population. Similarly, we arbitrarily selected four males and four females from the Ianthe (sexual) field-collected sample. We were unable to find any males in the several hundred adults from the sample collected from lake Sarah (suggesting that this sample is wholly asexual; also see [17]), and thus instead included four arbitrarily selected females from this sample. We followed the same procedures for four juvenile (<2 mm in shell length) snails from the Sarah field collection, with the exception that juvenile *P*. *antipodarum* cannot be reliably sexed (e.g., [22]). Because nearly all offspring produced by asexual polyploid female *P*. *antipodarum* are female [15], and because the lake Sarah population appears to be comprised only of triploid asexuals [17] (also see Table 1), it is likely (>92%, conservatively assuming that 2% of all triploids are male; [15]) that all of these Sarah-collected juveniles were female.

Each snail was sexed, with males distinguished from females by the presence of a penis. We then removed the shell and dissected each snail into “head” (anterior half, i.e., no reproductive tissue) and “body” (containing reproductive tissue along with digestive tissue) tissue subsamples because asexuality-causing endosymbionts such as *Wolbachia* typically reside in tissues associated with vertical transmission (e.g., [23]; reviewed in [9]; but see [24]). This dissecting approach allowed us to compare microbiome profiles in tissue with gonadal subcomponents to non-gonadal tissue. The head samples were dissected again in half, such that we then had three tissue samples that together encompassed one whole snail: two head halves and one body. Each tissue sample was then snap-frozen in liquid nitrogen and stored at -80°C until further processing. We dissected, extracted, and sequenced individual snails in three batches, each of which included both sexuals and asexuals and multiple asexual lineages, minimizing the potential that batch effects figured in the patterns we detected among our samples.

We next used flow cytometric analysis (following the protocol outlined in [17,21,25]) of the tissue from one head half per each of the field-collected snails to determine ploidy and thus infer reproductive mode of the samples [26]. As expected from earlier studies [17,21], all of the samples from Ianthe and Kaniere were diploid (sexual) and all of the samples from Sarah were triploid (asexual). Finally, we pooled tissue samples by sex and body section from three individuals in a single 1.5 mL Eppendorf tubes, such that each tube contained three tissue samples of the same type (head half *vs*. body) from each of three snails of the same sex, of the same ploidy level, and from the same population. While we pooled most tissue samples in order to ensure that we obtained enough DNA for sequencing, we did not pool tissue samples from the Ianthe and Sarah field collections (i.e., each sample contained the head or body tissue from one individual), which allowed us to investigate if any individual-level differences were detectable in *P*. *antipodarum* bacterial communities. Additional deviations from the standard protocol for the Sarah *P*. *antipodarum* included the use of only head tissue for the adults (no body tissue was available, which was used for another project) and the fact that juveniles cannot be sexed and can thus not be separated by sex. Because the Sarah juveniles were so small, we combined the remaining head half and body tissue for DNA extractions for each individual juvenile to ensure that we had enough tissue for DNA extraction. With the exception of these four juveniles, all samples were from adult snails. A detailed description of each sample is provided in Table 1 and S1 Table.

### DNA Sequencing and Analysis

We used the Qiagen DNeasy Plant kit (Qiagen, Valencia, CA, USA) following the manufacturer-specified protocol (with the exception of using nanopure water for elution) to extract DNA from each of the 60 tissue samples. Pyrosequencing was performed as described previously [27], using the universal bacterial primers 104F 5’-GGCGVACGGGTGAGTAA-3’ and 530R 5’-CCGCNGCNGCTGGCAC-3’ to target the V2-V3 region of the 16S rRNA gene [28]. For each sample, PCR was performed in triplicate with 100 ng of DNA by a single-step PCR to create 16S rRNA gene amplicons containing the Roche-specific sequencing adapters and a barcode unique to each sample. Amplicons were purified using Agencourt Ampure beads and combined in equimolar concentrations. Pyrosequencing was performed on a Roche 454 FLX instrument using Roche titanium reagents and procedures.

The 16S rRNA gene sequences were quality filtered, denoised, screened for PCR errors, and checked for chimeras using AmpliconNoise and Perseus to minimize potential methodological artifacts [29]. The Quantitative Insights into Microbial Ecology (QIIME) pipeline was used to analyze alpha and beta diversity of the bacterial DNA sequence data [30]. Unique operational taxonomic units (OTUs, i.e., DNA sequences or amplicon types) were identified by the 97% DNA identity criterion using the uclust OTU picker [31,32] in QIIME. A set of representative DNA sequences was chosen for each unique OTU in QIIME (pick_rep_set.py) and used for all subsequent analyses. Taxonomic affiliation was assigned to OTUs by comparing the rep-set DNA sequences to the Greengenes database (gg8.15.13 [33]). Rep-set DNA sequences were aligned using MUSCLE [32], and a phylogenetic tree necessary for the downstream alpha and beta diversity analysis was constructed using FastTree [34]. Measures of alpha diversity (Chao1, Shannon, dominance, equitability, Faith’s phylogenetic diversity [35], Good’s coverage) and beta diversity (Bray-Curtis and Unifrac distances [36]) were determined on a randomly selected subset of 400 sequences from each sample to standardize for varying sequencing effort across samples. Non-parametric t-tests with 1000 Monte Carlo permutations were used to determine if alpha diversity differed among the samples according to their reproductive mode (2x sexuals *vs*. pooled 3x and 4x asexuals) or other sample factors including lake of origin, population, population source (laboratory lineage or field-collected), sex, body section, or ploidy level. Bray-Curtis and weighted and unweighted Unifrac [36] distance matrices were generated, and principal coordinate analysis (PCoA) was performed as implemented in QIIME by the beta_diversity_through_plots.py script (-e 400). Select OTUs were compared to their nearest neighbor DNA sequence from Greengenes by aligning with MUSCLE, and a distance matrix was generated using the Jukes-Cantor model.

### Statistical Analysis

Statistical significance of community structure similarity was evaluated on Bray-Curtis distance matrices in the Community Analysis Package v.5 using analysis of similarity (ANOSIM). ANOSIM was performed on Unifrac distances in the vegan package [37] in R. In addition to testing individual sample factors, we nested samples within lake of origin when possible to control for variance due to origin from the same lake (e.g., for population, population source (laboratory lineage or field-collected), and body section). We were unable to control for lake of origin for reproductive mode or ploidy level because the *P*. *antipodarum* populations found in many of the lakes that we sampled represented only one or two ploidy levels and/or were entirely sexual or asexual.

We also investigated the degree to which bacterial community profiles identified sample factors by using random forests, a robust machine-learning technique for classification that is appropriate for a wide diversity of data types, including microbial community data [38]. We used the QIIME implementation of random forests with 10-fold cross-validation on a rarefied (-e 400) OTU level table (97% DNA identity). The OTU table was filtered before rarefaction to remove any OTUs that were observed in the dataset fewer than 10 times (using the filter_otus_from_otu_table.py script). SIMPER (Similarity Percentage) was used to determine the relative contribution of OTUs to the observed similarity/dissimilarity within sample type. Indicator Species Analyses (ISA; [39]) in PC-ORD (v 5.33) was used to determine which OTUs, if any, were statistically associated with reproductive mode and other sample factors and to confirm SIMPER results. Finally, the sensitivity of our results to specific lineages, sample types, bacterial phyla, and singletons was investigated by reanalyzing the original dataset after filtering samples (e.g., Ianthe snails, which comprised 57% of sexual snails) or specific OTUs (e.g., Cyanobacteria were likely an artifact of ingestion of cyanobacteria in New Zealand lakes (field-collected snails) and/or of our laboratory snail food, dried *Spirulina* cyanobacteria) from the data. Thus, we repeated all analyses described above after filtering the original OTU tables in QIIME (filter_samples_from_otu_table.py and filter_otus_from_otu_table.py).

### DNA Sequence Availability

All raw sequence data from this study are available through the NCBI Sequence Read Archive. The individual sff files from this study were assigned the accession numbers SAMN03276406 through SAMN03276465 under Bioproject PRJNA271685 (http://www.ncbi.nlm.nih.gov/bioproject/?term=PRJNA271685).

## Results

**Fig 1:**
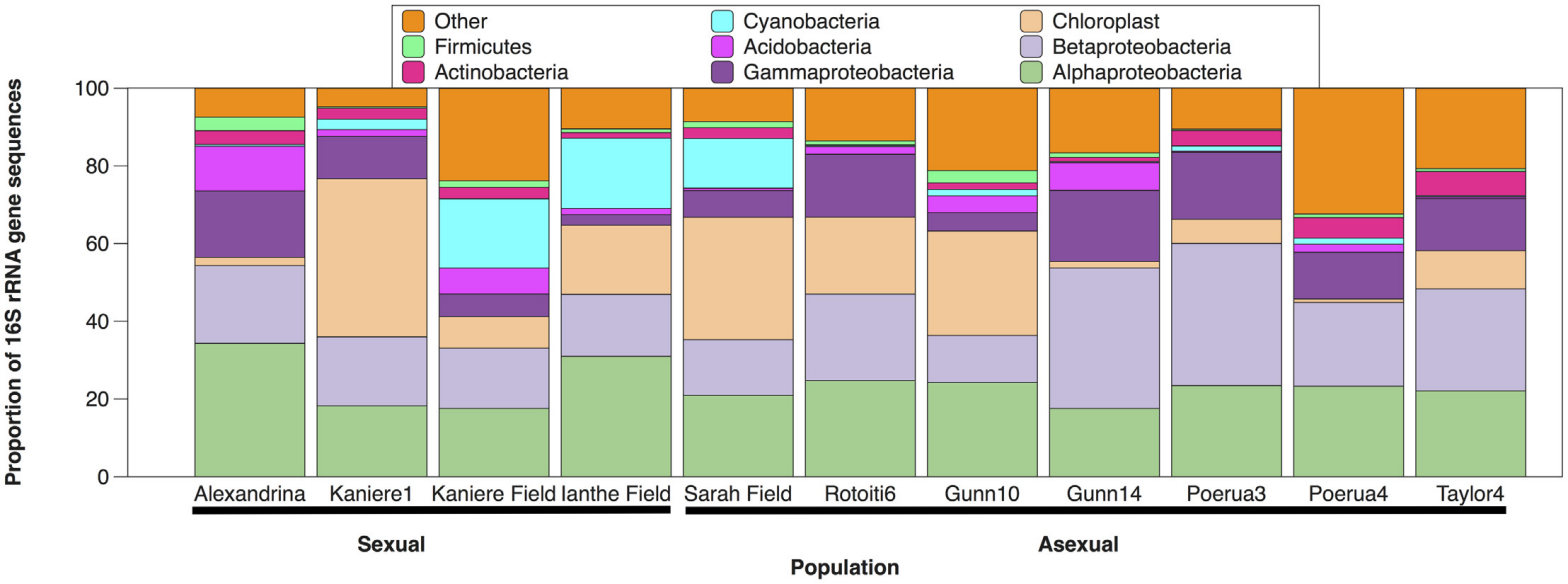
Phylum-level taxonomy of *P*. *antipodarum* populations expressed as the percentage of total sequences (prior to rarefaction and filtering Cyanobacteria OTUs). We detected 2624 unique OTUs (97% DNA similarity) distributed across approximately thirty phyla. Figure includes only the eight most abundant phyla; remaining OTUs are compressed into the “other” category.

Barcoded amplicon pyrosequencing targeting the bacterial 16S rRNA gene for the 60 *P*. *antipodarum* samples resulted in 258412 16S rRNA gene sequences. After quality filtering, denoising, and chimera removal, the mean number of sequences per sample was 4307 (range = 430 to 13333; SD = 3422). Good’s coverage estimates for the individual samples computed at an even depth of 400 sequences per sample ranged from 83% to 97% (mean = 90%; SD = 3%), indicating that a majority of the species richness that was PCR-amplified in the samples was detected in this DNA sequencing survey. We identified 2624 unique OTUs (97% DNA similarity) distributed across approximately thirty phyla (Fig 1 and S1 Fig). In general, samples were dominated by the Proteobacteria and Cyanobacteria, which comprised 58% and 23%, respectively, of the entire dataset. Proteobacteria sequences were primarily distributed among the alpha and beta classes (40% each), but were also represented by Gammaproteobacteria (10% of the Proteobacteria sequences). Sequences classified as Cyanobacteria were largely from chloroplast (66% of the cyanobacterial sequences) and likely represent algae inside snail intestinal tracts (*P*. *antipodarum* graze on algae growing on lake bottoms and vegetation surfaces and laboratory-cultured *P*. *antipodarum* are fed dried *Spirulina*, a cyanobacterium). Additional phyla detected among the samples include the Actinobacteria, Verrucomicrobacteria, Planctomycetes, and Firmicutes. Overall, bacterial communities varied among the individual samples; no OTUs were shared among all the samples, and only 11 of the total 2624 OTUs detected were shared among at least 75% of all samples (Table 2).

**Table 2:** Core OTUs across 75% of all 60 snail samples.

OTU	Taxonomic Assignment
96	Bacteria;Actinobacteria;Actinobacteria;Actinomycetales;Propionibacteriaceae;Propionibacterium
253	Bacteria; Proteobacteria; Alphaproteobacteria
362	Bacteria; Acidobacteria; Acidobacteria-6; iii1-15; mb2424
529	Bacteria; Proteobacteria; Alphaproteobacteria; Rhizobiales; Hyphomicrobiaceae
549	Bacteria; Proteobacteria; Alphaproteobacteria; Rhizobiales
608	Bacteria; Proteobacteria; Alphaproteobacteria; Sphingomonadales; Sphingomonadaceae
651	Bacteria; Proteobacteria; Betaproteobacteria; Burkholderiales; Comamonadaceae; Methylibium
726	Unknown Bacteria
969	Bacteria; Proteobacteria; Alphaproteobacteria; Rhodobacterales; Rhodobacteraceae; Rhodobacter
1813	Bacteria; Proteobacteria; Alphaproteobacteria; Rhizobiales; Hyphomicrobiaceae; Hyphomicrobium
2113	Bacteria; Proteobacteria; Betaproteobacteria; Burkholderiales; Comamonadaceae

Alpha diversity metrics used to estimate richness, dominance/equitability, phylogenetic diversity, and coverage among the samples were most variable when compared by lake, although the only significant differences detected for any pairwise lake comparisons were between the Chao1 estimates of Gunn vs. Ianthe samples and Alexandrina *vs*. Ianthe field collections (*p* = 0.028). In both cases, the Ianthe snails had significantly lower values of Chao1 diversity. In addition, dominance was significantly greater (*p* = 0.007) and equitability was significantly lower (*p* = 0.047) in the sexual snails compared to the asexual snails. No other significant alpha diversity differences were detected for any of the other sample factors (population source (laboratory lineage *vs*. field-collected), sex, body section, and ploidy level). Alpha diversity results summarized by sample factor are presented in S2 Table.

**Fig 2:**
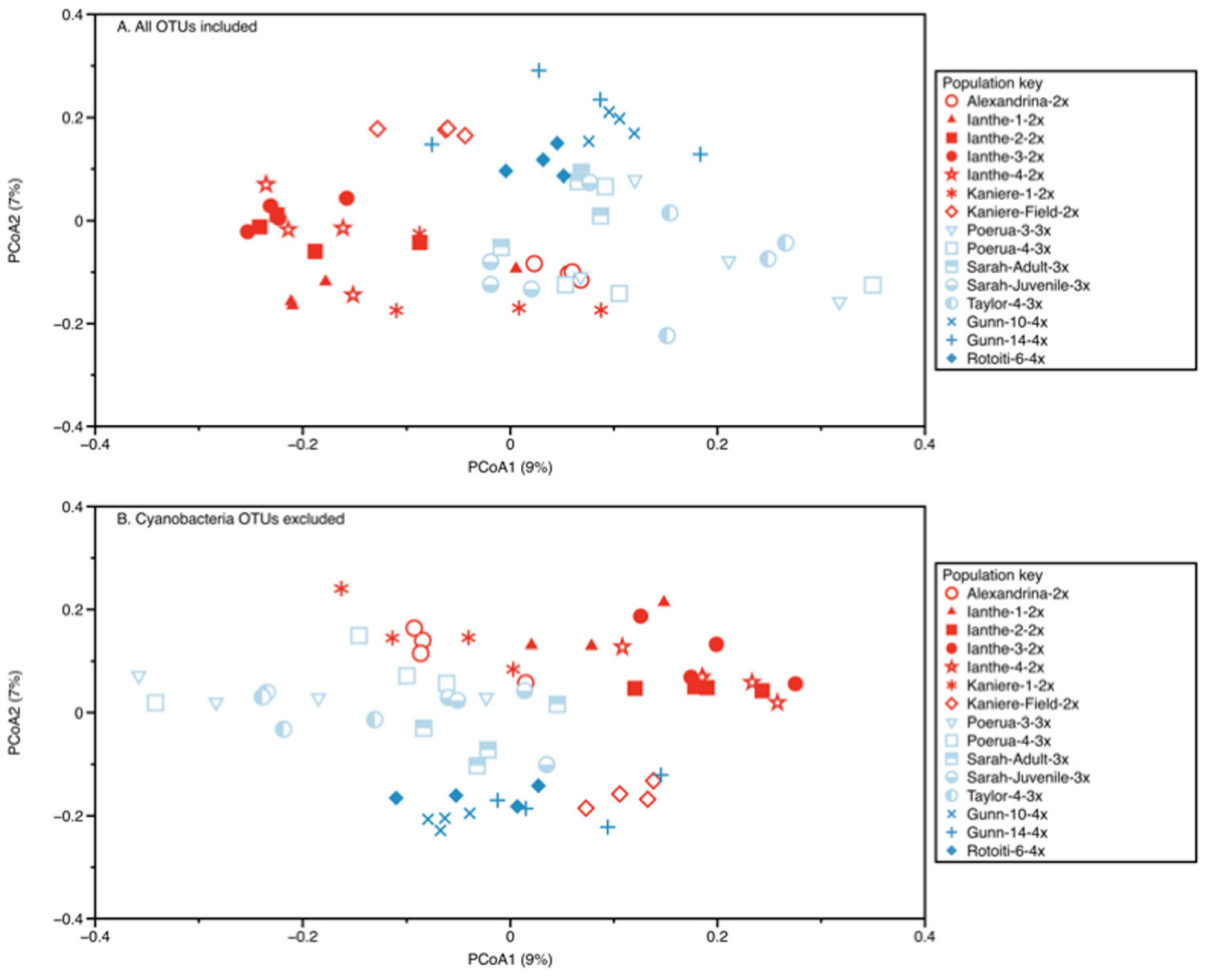
PCoA of unweighted Unifrac distances of bacterial communities associated with *P*. *antipodarum* determined from the entire dataset (panel A) and when Cyanobacteria OTUs were excluded from the analysis (panel B). The community composition and structural differences among the sexual (red symbols, 2x ploidy) and asexual (light blue open symbols, 3x ploidy; dark blue filled symbols, 4x ploidy) snails shown here were supported by ANOSIM (R-statistic = 0.274, *p* = 0.001). Within the adult asexual snails, the 3x samples were marginally different than the 4x samples (R-statistic = 0.114, *p* = 0.056). When Cyanobacteria OTUs were excluded, similarly significant or even stronger clustering was observed among the samples (R-statistic = 0.240, *p* = 0.001 for reproductive mode and R-statistic = 0.260, *p* = 0.001 for ploidy). Lake and population were the only factors to explain as much or more variance in snail bacterial community structure as reproductive mode.

There were significant differences in bacterial community composition and structure between sexual and asexual *P*. *antipodarum* (Fig 2A): Sexual and asexual snail samples tended to cluster separately in PCoA ordinations of distance matrices based on OTU presence/absence (unweighted Unifrac) and abundance (Bray-Curtis and weighted Unifrac). Community composition and structural differences among the sexual and asexual snails were supported by ANOSIM (Bray-Curtis R-statistic = 0.274, *p* = 0.001). Within the adult asexual snails, the 3x samples were marginally different than the 4x samples (Bray-Curtis R-statistic = 0.114, *p* = 0.056), though the global R-statistic for ploidy was not significant for Bray-Curtis distances. We did observe significant clustering of samples by ploidy among the Unifrac distances (ANOSIM R-statistic for unweighted Unifrac = 0.228 and weighted Unifrac = 0.149, *p* = 0.001 for both unweighted and weighted Unifrac distances), reinforcing the potential that these analysis results do reflect ploidy effects.

Because the Ianthe snails represented over half of the sexual samples (16/28; ~57%), it is possible that Ianthe-specific effects rather than sex *per se* are driving the significant differences between sexual and asexual-associated bacterial communities. We addressed this possibility by removing the Ianthe snails from the analysis, which revealed that samples continued to cluster by ploidy (S2 Fig, Bray-Curtis R-statistic = 0.219, *p* = 0.002), though the ANOSIM R-statistic (0.09) of the Bray-Curtis distance for reproductive mode was not significant (*p* = 0.102). One potential explanation for our failure to detect a reproductive mode effect on clustering in the Ianthe-filtered dataset is that removal of the 16 Ianthe samples meant that our reproductive mode-focused comparisons were now imbalanced and suffering from low statistical power, with only 12 sexual samples relative to the 32 asexual samples. The likelihood of this explanation is reinforced by a power analysis of the mean distances between samples within the same reproductive mode compared to samples from different reproductive modes using a Mann-Whitney test of two independent samples (non-parametric; performed in XLSTAT v.2016.01.26437), which indicated that for an alpha of 0.05 and a sample size of 562 observations, the type 2 error is 0.949 and the power is 0.051. Significant clustering by both reproductive mode and ploidy were also observed for Unifrac distances (*p* <0.03 for weighted and unweighted distances). Taken together, these results at least suggest that the reproductive mode and ploidy effects we have observed are not driven entirely by the Ianthe samples.

All significant differences among pairwise comparisons of reproductive mode by sex in the original dataset were between sexual and asexual snails; there were no significant differences between males and females within the same reproductive modes (S3 Table). We did detect significant differences in the bacterial communities of head vs. body tissue in the sexual snails (Bray-Curtis R-statistic = 0.111, *p* = 0.03, S3 Table), but not within the asexual snails (*p* = 0.476). There was significant clustering by other sample factors (Table 3), but lake (Bray-Curtis R-statistic = 0.389, *p* = 0.001) and population (Bray-Curtis R-statistic = 0.716, *p* = 0.001) were the only factors to explain as much or more variation in snail bacterial community structure as reproductive mode. Similar trends were observed for all factors of ANOSIM results of Unifrac distances, but global R-statistics were always greater than the R-statistics for Bray-Curtis distances. Thus, the ANOSIM results we report for the Bray-Curtis distances are conservative estimates of the relationships we observed among snail bacterial community composition and sample factors.

**Table 3:** Analysis of similarity (ANOSIM) of Bray-Curtis distances among snail bacterial communities by sample factor.

Sample Factor	Factor Groups	Global R-statistic^a^	*p* value
**Reproductive mode**			
	Sexual *vs*. Asexual	0.274	0.001
**Ploidy level**			
	2x *vs*. 3x *vs*. 4x	0.083	0.058
**Lake**			
	Gunn *vs*. Taylor *vs*. Ianthe etc.	0.389	0.001
**Population**			
	Gunn10 *vs*. Gunn14 *vs*. Ianthe etc.	0.716	0.001
**Source**			
	Laboratory lineage *vs*. field-caught	0.154	0.095
**Sex**			
	Male *vs*. Female	0.002	0.434
**Body section**			
	Head *vs*. Body	0.089	0.013

^a^Significant within-factor pairwise comparisons within sample factor are given in S2 Table (Reproductive mode) and S3 Table (Population).

We investigated bacterial community composition variation among individuals of the same lineage by comparing a set of the Ianthe samples that were not pooled before PCR amplification, but did not detect significant differences among ANOSIM pairwise comparisons (*p* = 0.128–0.728). This result suggests that individual snail differences do not contribute significantly to factor differences. We did find that juvenile snails from Sarah contained significantly different communities than adults from the same lake (Sarah R-statistic = 0.916, *p* = 0.014). Because the juvenile samples from Sarah included pooled heads and bodies while the adult samples from Sarah only included heads, we cannot exclude the possibility that this difference between lake Sarah adults and juveniles may be attributed to the tissue type used for DNA extraction.

Despite being maintained under identical conditions, there was significant variation in the bacterial communities among the laboratory lineages (ANOSIM R-statistic = 0.716, *p* = 0.001). This effect extended even to lineages that originated from the same lake but were maintained in separate tanks (e.g., the two replicate lineages from lake Gunn, R-statistic = 1, *p* = 0.014). Although global R-statistics did not indicate that laboratory lineages harbored significantly different bacterial communities than field-collected snails (R-statistic = 0.154, *p* = 0.095), the Kaniere laboratory-cultured snail communities were significantly different than the Kaniere field-collected snails (R-statistic = 0.989, *p* = 0.014). ANOSIM results by population are summarized in S4 Table.

We also used the classification algorithm random forests to test how well the bacterial communities we detected predicted sample factors. ANOSIM can sometimes fail to detect significant clustering among samples in studies with an unbalanced design and heterogeneous distances among groups [40] or when gradients in community composition exist among groups [41]. In general, distance-based tests such as ANOSIM can confound community composition differences among samples, depending on dispersion homogeneity among factors and the relative location of the centroid of the sample clusters [42,43]. We were particularly interested in confirming factors with low ANOSIM R-statistics (<0.400) but significant *p* values (e.g., reproductive mode and ploidy level).

Overall, random forests results were similar to ANOSIM; factors related to lake, population, reproductive mode, and ploidy level were the strongest predictors of community composition, whereas a sample’s source (field vs. laboratory), sex, and body section were much weaker factors (Table 4). Ratios of random error to model error were 29.33 and 66 for lake and population, respectively (a minimum ratio of 2 is expected for factors that can be accurately predicted [38]). For reproductive mode and ploidy level of the samples, the ratios of random error to model error were 14 and 16, respectively. The predictive accuracy was 95% to 99% for lake, population, reproductive mode, and ploidy level (only 1–3 of 60 samples were misclassified), whereas the predictive accuracy of the sample’s source, sex, and body section ranged from 30% to 92% and had correspondingly low ratios of random error to model error (0.7 to 2.37). Thus, random forest classifiers indicated that source, sex, and body section are poor predictors of snail community composition, compared to factors related to lake and reproductive mode.

**Table 4:** Results from random forests classifiers.

Factors	Ratio^a^	Predictive Accuracy (%)
Sex	0.7	30
Section	1.16	55
Source	2.37	92
Reproductive Mode	14	97
Ploidy	16	97
Population	29.33	95
Lake	66	99

^a^Ratio of random error to model error.

The mean dissimilarity of the bacterial communities between the sexual and asexual samples was 90% (SIMPER analysis, Table 5). We identified twenty OTUs that cumulatively accounted for 50% of the community compositional differences between sexual and asexual snails. The three OTUs that explained the most variance between sexual and asexual snails were identified as Cyanobacteria (2 OTUs) and a member of the Alphaproteobacteria. These three OTUs were most abundant in the sexual snails, specifically the head section, which differed from asexual heads (ANOSIM R-statistic = 0.345, *p* = 0.001) and sexual bodies (ANOSIM R-statistic = 0.111, *p* = 0.03). The Alphaproteobacterium OTU is a sequence that was classified as a member of the Rickettsiales by QIIME, which uses a limited reference database. In order to provide a more specific taxonomic description of this sequence, we used blastn against the NCBI non-redundant nucleotide (nr) database (a much larger sequence database than QIIME), to find that the DNA sequence of this OTU is 99% similar (over 388 nucleotides) to various 16S rRNA gene sequences in Genbank from uncultured organisms originated from aquatic and soil environments. These taxa include several Rickettsiales members, including some Rickettsiales that were affiliated with putative endosymbionts of Acanthomoeba according to their sequence metadata in Genbank.

**Table 5:** Similarity percentage analysis (SIMPER) between sexual and asexual snails. Mean dissimilarity between sexual and asexual snails = 90%.

OTU	Mean abundance in sexual snails	Mean abundance in asexual snails	Mean Dissimilarity	% Contribution	Cumulative %	Taxonomic Assignment (quality score)^a^
**Dominant in sexual snails**						
2137	649.79	218.25	7.67	8.48	8.48	Bacteria;Cyanobacteria (1.0)
2283	514.39	62.31	5.63	6.22	14.7	Bacteria;Cyanobacteria;Chloroplast;Rhodophyta (1.0)
985	529.89	0.16	5.15	5.69	20.39	Bacteria;Proteobacteria;Alphaproteobacteria;Rickettsiales (0.96)
2302	341.82	6.47	3.19	3.53	28.07	Bacteria;Cyanobacteria;Chloroplast;Stramenopiles (1.0)
270	272	21.5	2.58	2.85	30.92	Bacteria;Proteobacteria;Betaproteobacteria;Burkholderiales;Comamonadaceae (1.0)
549	157.57	56.81	1.81	2	37.63	Bacteria;Proteobacteria;Alphaproteobacteria;Rhizobiales (0.96)
182	60.14	38.06	0.96	1.06	46.8	Bacteria;Planctomycetes;OM190;CL500-15 (1.0)
362	72.82	41.22	0.91	1.01	47.8	Bacteria;Acidobacteria;Acidobacteria-6;iii1-15;mb2424 (0.99)
228	76.32	23.81	0.89	0.98	49.79	Bacteria;Proteobacteria;Betaproteobacteria;Burkholderiales;Comamonadaceae;Rubrivivax (0.98)
**Dominant in asexual snails**						
1972	11.54	375.47	3.76	4.16	24.54	Bacteria;Cyanobacteria;Chloroplast;Stramenopiles (1.0)
2217	0.14	314.19	2.33	2.58	33.49	Bacteria;Cyanobacteria;Chloroplast;Stramenopiles (0.99)
1691	4	251.09	1.93	2.13	35.63	Bacteria;Proteobacteria;Alphaproteobacteria;Rhodobacterales;Rhodobacteraceae;Rhodobacter (1.0)
821	8.43	96.41	1.52	1.68	39.31	Bacteria;Proteobacteria;Gammaproteobacteria;Alteromonadales;[Chromatiaceae];Rheinheimera (1.0)
1014	48.82	72.31	1.31	1.45	40.76	Bacteria;Proteobacteria;Gammaproteobacteria;Pseudomonadales;Pseudomonadaceae (1.0)
969	54.5	79.28	1.29	1.43	42.19	Bacteria;Proteobacteria;Alphaproteobacteria;Rhodobacterales;Rhodobacteraceae;Rhodobacter (1.0)
104	2.93	82.38	1.08	1.2	43.39	Bacteria;Proteobacteria (0.99)
1327	19.25	49.72	1.07	1.18	44.56	Bacteria;Proteobacteria;Alphaproteobacteria;Caulobacterales;Caulobacteraceae;Phenylobacterium (1.0)
2405	0	70.16	1.06	1.18	45.74	Bacteria;Proteobacteria;Betaproteobacteria (0.85)
634	7.68	40.69	0.91	1.01	48.81	Bacteria;Proteobacteria;Betaproteobacteria;Burkholderiales;Comamonadaceae (1.0)

^a^Consensus taxonomic assignment by assign_taxonomy.py in QIIME (-m uclust), quality scores represents the proportion of sequences that match the assignment.

The three OTUs that were the most enriched in asexual vs. sexual snails were also assigned to the Cyanobacteria and Alphaproteobacteria. Rather than being affiliated with the Rickettsiales, the Alphaproteobacterium OTU from the asexual snails was classified as a Rhodobacteriales by QIIME and was 99% similar to uncultured *Rhodobacter* DNA sequences in Genbank. We confirmed that the abundance of singletons in our OTU table (94% of the cells were empty) was not obscuring any patterns in our data by repeating our analyses on an OTU table that had been filtered for rare OTUs (any OTUs that were represented by <25 sequences). Overall, results were unchanged by this filtering process, although the difference between sexual and asexual snails calculated by SIMPER decreased by 3% (from 90% to 87%). Random forests analysis also confirmed the importance of the SIMPER OTUs in sexual compared to asexual samples, and results from the indicator species analysis were qualitatively similar to the outcome of the SIMPER analysis. For example, Cyanobacteria and Alphaproteobacteria still contributed significantly to observed differences associated with reproductive mode in the indicator species analysis, although there were some differences in the specific OTUs that differed between sexual and asexual snails in these two analyses (Table 6).

**Table 6:** Indicator species analysis by reproductive mode.

Reproductive Mode	OTU	Indicator Value	*p* value	Taxonomic Assignment (quality score)^a^
**Sexual**	1813	83.5	0.0002	Bacteria;Proteobacteria;Alphaproteobacteria;Rhizobiales;Hyphomicrobiaceae;Hyphomicrobium (0.94)
	2302	80.6	0.0002	Bacteria;Cyanobacteria;Chloroplast;Stramenopiles (1.0)
	270	76.1	0.0002	Bacteria;Proteobacteria;Betaproteobacteria;Burkholderiales;Comamonadaceae (1.0)
	726	74.6	0.0002	Bacteria (1.0)
	1634	74.4	0.0002	Bacteria;Proteobacteria;Alphaproteobacteria;Rhodobacterales;Hyphomonadaceae (0.98)
	1091	67.2	0.0002	Bacteria;Proteobacteria;Alphaproteobacteria;Sphingomonadales (1.0)
	2161	52.4	0.0002	Bacteria;Actinobacteria;Actinobacteria;Actinomycetales (0.85)
	659	46.4	0.0002	Bacteria;Proteobacteria (0.90)
	1322	46	0.0002	Bacteria;Proteobacteria;Alphaproteobacteria;Sphingomonadales (1.0)
	1798	61.7	0.0004	Bacteria;Proteobacteria;Betaproteobacteria;Burkholderiales;Burkholderiaceae (0.91)
	1891	42.5	0.0004	Bacteria;Bacteroidetes;Flavobacteriia;Flavobacteriales;Cryomorphaceae (0.89)
	985	39.3	0.0004	Bacteria;Proteobacteria;Alphaproteobacteria;Rickettsiales (0.96)
	160	35.5	0.0004	Bacteria;Cyanobacteria;Synechococcophycideae;Synechococcales (0.95)
	608	71.8	0.0008	Bacteria;Proteobacteria;Alphaproteobacteria;Sphingomonadales;Sphingomonadaceae (0.91)
	2388	65.7	0.0008	Bacteria;Proteobacteria;Betaproteobacteria;Burkholderiales;Burkholderiaceae (0.90)
**Asexual**	1691	86.1	0.0002	Bacteria;Proteobacteria;Alphaproteobacteria;Rhodobacterales;Rhodobacteraceae;Rhodobacter (1.0)
	1972	81.9	0.0002	Bacteria;Cyanobacteria;Chloroplast;Stramenopiles (1.0)
	2217	81.2	0.0002	Bacteria;Cyanobacteria;Chloroplast;Stramenopiles (0.99)
	2405	81.2	0.0002	Bacteria;Proteobacteria;Betaproteobacteria (0.85)
	1494	59	0.0002	Bacteria;Actinobacteria;Actinobacteria;Actinomycetales;Actinomycetaceae;Actinomyces (1.0)
	1483	50	0.0002	Bacteria;Proteobacteria;Betaproteobacteria;Methylophilales;Methylophilaceae (1.0)
	2075	49.7	0.0002	Bacteria;Proteobacteria;Deltaproteobacteria;Myxococcales (0.98)
	941	43.7	0.0002	Bacteria;Bacteroidetes;Sphingobacteriia;Sphingobacteriales;Saprospiraceae (1.0)
	821	77.6	0.0004	Bacteria;Proteobacteria;Gammaproteobacteria;Alteromonadales;[Chromatiaceae];Rheinheimera (1.0)
	347	37.5	0.0004	Bacteria;WPS-2 (0.97)
	516	42.9	0.0006	Bacteria;Proteobacteria;Betaproteobacteria;Burkholderiales;Comamonadaceae;Limnobacter (1.0)
	48	42.6	0.0008	Bacteria;Verrucomicrobia;Verrucomicrobiae;Verrucomicrobiales;Verrucomicrobiaceae;Prosthecobacter;debontii (0.91)

^a^Consensus taxonomic assignment by assign_taxonomy.py in QIIME (-m uclust), quality scores represent the proportion of sequences that match the assignment.

**Fig 3:**
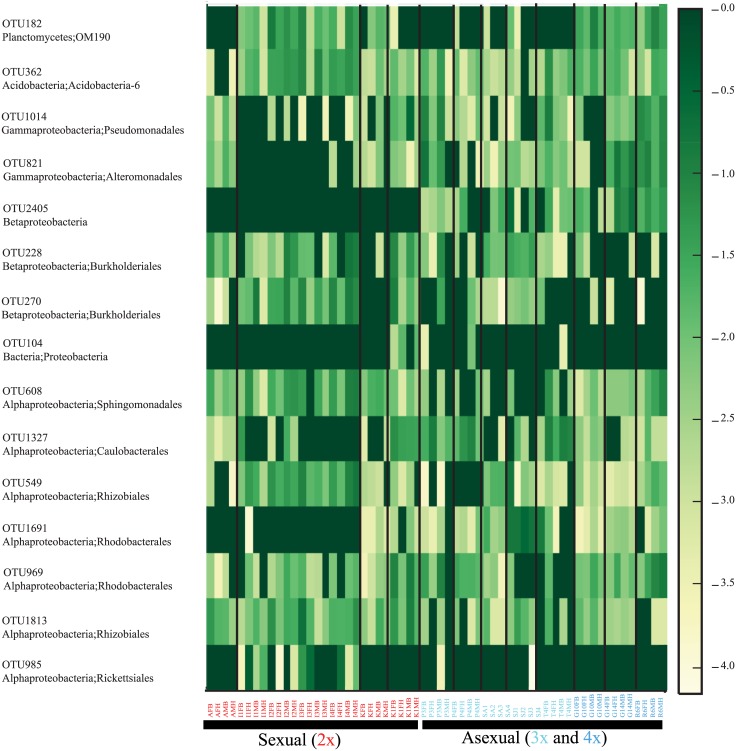
Heatmap showing relative abundance of non-Cyanobacteria OTUs identified by SIMPER as contributing significantly to the dissimilarity between sexual and asexual snails. Vertical lines delineate individual populations. Samples were normalized and relative abundance of each OTU was log transformed. Color legend on right indicates the relative abundance of each OTU where lighter colors indicate greater OTU abundance.

The importance of the Cyanobacteria OTUs in the SIMPER and Indicator Species analysis is concerning because we suspect that these sequences are derived from snail food and are thus unlikely to be involved in reproductive mode. Therefore, we performed all analyses described above with an OTU table that was filtered to remove any OTUs assigned to the phylum Cyanobacteria. Removal of these sequences did not qualitatively change any of the results described above, but did result in similarly significant or even stronger clustering among the samples (Fig 2B, R-statistic = 0.240, *p* = 0.001 for reproductive mode; R-statistic = 0.260, *p* = 0.001 for ploidy; Bray-Curtis distances). The non-cyanobacterial indicator taxa detected by SIMPER were also the same taxa revealed by analyses of the original complete dataset. All OTUs identified by SIMPER (before and after filtering Cyanobacteria DNA sequences) were unique (<97% similar to each other in their DNA sequence) and were 64 to 100% similar to their nearest relatives identified from the NCBI nr database. A distance matrix comparing the genetic distance among the OTUs identified by SIMPER and their nearest relatives in Genbank is given in S5 Table. A heatmap showing the relative abundance of the SIMPER OTUs across all samples highlights the differences among factors (Fig 3), including the absence of the Rickettsiales OTU from nearly all of the asexual snail samples and the presence of some OTUs (e.g., OTU2405, an unassigned Betaproteobacteria) that were strictly limited to the asexual snails. All of these results must formally be considered from the perspective of contamination, which cannot be ruled out in any study using sensitive techniques such as PCR and next-generation sequencing. Because all of our samples were treated similarly, we would expect any contaminating DNA sequence to manifest in our dataset as low-abundance OTUs distributed uniformly across all samples. The results we report here do not represent this type of pattern, allowing us to conclude that contamination is not likely to be a major driver of any of our findings.

## Discussion

Microbes that reside in the animal body have the potential to play an important role in host reproduction. Here, we found evidence suggesting distinct differences in the microbiome composition between sexual and asexual freshwater snails sampled from multiple laboratory lineages and New Zealand lakes. In particular, the sexual and asexual *P*. *antipodarum* included in our study differed with respect to the presence of Rickettsiales bacteria in the somatic tissue of sexual individuals and *Rhodobacter* in asexual individuals.

We also detected strong phylogeographic signatures in the bacterial community structure associated with snails from populations from different lakes and from different populations within lakes. These results mirror the outcomes of phylogeographic analyses of *P*. *antipodarum* itself, which reveals substantial genetic variation among *P*. *antipodarum* of the same ploidy level and reproductive mode but from different lakes, as well as among *P*. *antipodarum* that differ in ploidy level and reproductive mode [21]. Because the comparisons between sexual and asexual snails required samples from different populations and different lakes, and because we could not control for phylogenetic nonindependence, it is possible that the significant differences in microbiota community structure between 2x, 3x, and 4x snails revealed by the principal coordinate analysis could be due to a population and/or lake effect rather than a reproductive mode effect *per se*. Even so, the community compositional differences between samples of different ploidy levels (Fig 2) suggest that the effects of reproductive mode that we report here are not a simple artifact of phylogeography. For example, the variance between the bacterial communities harbored by sexual *vs*. asexual populations (ANOSIM R-statistic = 0.274, *p* = 0.001) was more than two-fold greater than between the bacterial communities harbored by triploid and tetraploid populations (ANOSIM R-statistic = 0.114, *p* = 0.056), despite the fact that all four groupings (sexual, asexual, triploid, tetraploid) represented populations from multiple lakes. While teasing apart the effects of ploidy from population in *P*. *antipodarum* will require additional study (and in particular, the inclusion of more populations), that the 3x and 4x snails in our study did possess different microbiota is intriguing in light of the distinct possibility that ploidy level can influence immune function and host resistance [44].

The case for a connection between reproductive mode and microbiome community in sexual *P*. *antipodarum* is strengthened by the absence of Rickettsiales in the triploid and tetraploid asexuals and their presence in both sexual males and females (Fig 3), regardless of lake origin. Members of the group Rickettsiales have a wide host range and cause a diversity of host effects across the parasite-mutualist continuum [45]. These symbionts can be transmitted through blood-sucking arthropods such as ticks and are commonly found as parasitic bacteria in herbivorous arthropods [46] and vertebrates [45]. Rickettsiales have also been found associated with established non-arthropod hosts like leeches and amoeba [47,48], though their effects are largely unknown. Here, the presence of Rickettsiales in both male and female sexual *P*. *antipodarum* from both field-collected and laboratory-cultured sexual individuals (see Fig 3) hints at its effects and transmission routes. In particular, the first result suggests that males inherit or acquire Rickettsiales in a similar manner to females, while the second result suggests that these symbionts are persistent and might be vertically transmitted within sexual lineages. The apparent absence of Rickettsiales from the body tissue of some of the sexual *P*. *antipodarum* studied here could highlight a role for horizontal transmission or suggest that symbionts develop in one part of the body before transferring to germ tissue [24]. As suggested by Frost et al. [24], a tropism in non-reproductive tissue may rule out any specialization for reproductive parasitism (e.g., sex-ratio distortion, cytoplasmic incompatibility, parthenogenesis induction) and suggest alternative parasitic or even mutualistic phenotypic effects.

Across populations of field collections and laboratory-cultured lineages, asexual snails consistently possessed bacteria closely related to the Proteobacteria genus *Rhodobacter* in their microbiome. *Rhodobacter* bacteria are phototrophic in aquatic environments and have been found to be symbionts of marine sponges [49] and *Daphnia* [50], although their symbiotic effects remain unknown. The transmission route of *Rhodobacter* bacteria into *P*. *antipodarum* individuals is also not clear, and may only reflect their abundance in aquatic environments. The fact that *Rhodobacter* was found in both adults and juveniles from Lake Sarah (and at a much higher abundance in juveniles) suggests that this bacterium might be an inherited endosymbiont. Several established endosymbiotic bacteria belonging to the genera *Burkholderia*, *Aeromonas*, *Brevibacillus*, and *Ideonella* were present in both adults and juveniles from lake Sarah, indicating that some or all of these bacteria might also be inherited or acquired soon after birth. Additional comparisons of more juveniles and adults from additional populations will be required to more fully evaluate this possibility.

Genetic differentiation between host strains has been implicated in the variation of the microbiota associated with laboratory-reared animals [51,52]. We used comparisons among genetically distinct asexual populations descended from snails originally collected from Lakes Taylor, Rotoiti, Poerua, and Gunn and maintained in the laboratory over several generations to detect multiple genotype-specific effects on microbiome composition. Our results indicate that these different *P*. *antipodarum* populations are associated with different bacterial communities even when raised under common garden conditions. Previous studies of the bacterial communities of various *Drosophila* species indicated that there is a large difference in the diversity and community composition of laboratory lineages *vs*. wild populations of this important model organism. Given these discrepancies, it might be difficult to justify the use of laboratory cultures as appropriate models of host—microbe interactions that occur in the wild [53]. By contrast, we found no significant difference in the diversity or community composition between our laboratory lineages and field-caught populations, except between the Kaniere samples. While this laboratory/field comparison requires more rigorous sampling and testing to be considered definitive, the lack of evidence for markedly different bacterial communities between our laboratory and field-collected snails raises the interesting possibility that *P*. *antipodarum* has the potential to be a good model for the laboratory study of host-microbe interactions *in situ*.

One particularly exciting potential implication of intraspecific variation in *P*. *antipodarum* microbiota communities is the possibility that across-lineage microbial variation could mediate the already well-documented coevolutionary interactions between *P*. *antipodarum* and *Microphallus*, a sterilizing trematode worm [13,54–56]. Microbial host-protection against infection has been demonstrated across animal and plant species [57,58]. In bumblebees, genotype-specific microbial communities have been shown to facilitate infection specificity [59]. Infection by *Microphallus* in lake populations of *P*. *antipodarum* snails is host genotype-specific [20,60], hinting that the genotype-specific variation in the microbiome could play a role in mediating this host-parasite interaction.

Altogether, multiple lines of analyses suggest that lake, population, ploidy level, and reproductive mode might be linked to differences in bacterial community structure of a diverse array of sexual and asexual *P*. *antipodarum*. Although we do not yet know the exact nature of these associations, the consistent differences in Rickettsiales and *Rhodobacter* presence between sexual and asexual *P*. *antipodarum* set the stage for future studies that more directly address whether these microbes play a role in host reproduction. Critical next steps in addressing this possibility include experimental manipulations aimed at determining whether exposure to and elimination of particular bacterial taxa can influence *P*. *antipodarum* reproductive mode (e.g., [61]) as well as direct comparisons of the bacterial communities of sexual and asexual *P*. *antipodarum* originating from the same New Zealand lakes.

## Supporting Information

S1 Fig Barchart showing the percent composition of OTUs identified at the 97% DNA identity level across all samples included in this study. Only OTUs contributing more than 2% of total DNA sequence data are annotated in the legend, which includes all non-cyanobacterial OTUs identified by SIMPER.
(EPS)

S2 Fig PCoA of unweighted Unifrac distances of bacterial communities associated with *P*. *antipodarum* from 10 of the populations used in this study. Ianthe samples, which comprised 57% of the 2x samples, are not included in this analysis to determine if Ianthe snails were driving sexual vs. asexual clustering patterns observed in the entire dataset. Cyanobacteria OTUs were not filtered. Sexual samples are in red and asexual samples are in blue. Ploidy (2x, 3x, 4x) of each population is given in the legend.
(EPS)

S1 Table List and description of samples. (PDF)

S2 Table Summary of alpha diversity results and non-parametric t-test with 1000 Monte Carlo permutations. (PDF)

S3 Table Analysis of similarity (ANOSIM) of Bray-Curtis distances among snail bacterial communities by reproductive mode. (PDF)

S4 Table Analysis of similarity (ANOSIM) of Bray-Curtis distances among snail bacterial communities by snail population. (PDF)

S5 Table Estimates of 16S rRNA gene divergence between OTU sequences identified as significant in SIMPER and nearest BLAST matches. (PDF)
