# Supplementary material for: Distinct Bacterial Microbiomes in Sexual and Asexual Potamopyrgus antipodarum, a New Zealand Freshwater Snail
Source: PLoS One. 2016 Aug 26;11(8):e0161050. doi: 10.1371/journal.pone.0161050 (PMC5001651; doi:10.1371/journal.pone.0161050)
Supplement: S1 Table — (PDF) [file pone.0161050.s003.pdf]

S1 Table. List and description of samples.

SampleID	Lake	Replicate	Pooled or Individual Snail	Sex	Ploidy	Inferred Reproductive Mode	Section	Source
AFB	Alexandrina	Alexandrina	Pooled	Female	2X	Sexual	Body	LL
AFH	Alexandrina	Alexandrina	Pooled	Female	2X	Sexual	Head	LL
AMB	Alexandrina	Alexandrina	Pooled	Male	2X	Sexual	Body	LL
AMH	Alexandrina	Alexandrina	Pooled	Male	2X	Sexual	Head	LL
I1FB	Ianthe	Ianthe-1	Individual	Female	2X	Sexual	Body	Field
I1FH	Ianthe	Ianthe-1	Individual	Female	2X	Sexual	Head	Field
I1MB	Ianthe	Ianthe-1	Individual	Male	2X	Sexual	Body	Field
I1MH	Ianthe	Ianthe-1	Individual	Male	2X	Sexual	Head	Field
I2FB	Ianthe	Ianthe-2	Individual	Female	2X	Sexual	Body	Field
I2FH	Ianthe	Ianthe-2	Individual	Female	2X	Sexual	Head	Field
I2MB	Ianthe	Ianthe-2	Individual	Male	2X	Sexual	Body	Field
I2MH	Ianthe	Ianthe-2	Individual	Male	2X	Sexual	Head	Field
I3FB	Ianthe	Ianthe-3	Individual	Female	2X	Sexual	Body	Field
I3FH	Ianthe	Ianthe-3	Individual	Female	2X	Sexual	Head	Field
I3MB	Ianthe	Ianthe-3	Individual	Male	2X	Sexual	Body	Field
I3MH	Ianthe	Ianthe-3	Individual	Male	2X	Sexual	Head	Field
I4FB	Ianthe	Ianthe-4	Individual	Female	2X	Sexual	Body	Field
I4FH	Ianthe	Ianthe-4	Individual	Female	2X	Sexual	Head	Field
I4MB	Ianthe	Ianthe-4	Individual	Male	2X	Sexual	Body	Field
I4MH	Ianthe	Ianthe-4	Individual	Male	2X	Sexual	Head	Field
K1FB	Kaniere	Kaniere-1	Pooled	Female	2X	Sexual	Body	Field
K1FH	Kaniere	Kaniere-1	Pooled	Female	2X	Sexual	Head	Field
K1MB	Kaniere	Kaniere-1	Pooled	Male	2X	Sexual	Body	Field
K1MH	Kaniere	Kaniere-1	Pooled	Male	2X	Sexual	Head	Field
KFB	Kaniere	Kaniere	Pooled	Female	2X	Sexual	Body	LL
KFH	Kaniere	Kaniere	Pooled	Female	2X	Sexual	Head	LL
KMB	Kaniere	Kaniere	Pooled	Male	2X	Sexual	Body	LL
KMH	Kaniere	Kaniere	Pooled	Male	2X	Sexual	Head	LL
P3FB	Poerua	Poerua-3	Pooled	Female	3X	Asexual	Body	LL
P3FH	Poerua	Poerua-3	Pooled	Female	3X	Asexual	Head	LL
P3MB	Poerua	Poerua-3	Pooled	Male	3X	Asexual	Body	LL
P3MH	Poerua	Poerua-3	Pooled	Male	3X	Asexual	Head	LL
P4FB	Poerua	Poerua-4	Pooled	Female	3X	Asexual	Body	LL
P4FH	Poerua	Poerua-4	Pooled	Female	3X	Asexual	Head	LL
P4MB	Poerua	Poerua-4	Pooled	Male	3X	Asexual	Body	LL
P4MH	Poerua	Poerua-4	Pooled	Male	3X	Asexual	Head	LL

SA1	Sarah	Sarah-Adult-1	Individual	Female	3X	Asexual	Adult	Field
SA2	Sarah	Sarah-Adult-2	Individual	Female	3X	Asexual	Adult	Field
SA3	Sarah	Sarah-Adult-3	Individual	Female	3X	Asexual	Adult	Field
SA4	Sarah	Sarah-Adult-4	Individual	Female	3X	Asexual	Adult	Field
SJ1	Sarah	Sarah-Juvenile-1	Individual	Juvenile	3X	Asexual	Juvenile	Field
SJ2	Sarah	Sarah-Juvenile-2	Individual	Juvenile	3X	Asexual	Juvenile	Field
SJ3	Sarah	Sarah-Juvenile-3	Individual	Juvenile	3X	Asexual	Juvenile	Field
SJ4	Sarah	Sarah-Juvenile-4	Individual	Juvenile	3X	Asexual	Juvenile	Field
T4FB	Taylor	Taylor-4	Pooled	Female	3X	Asexual	Body	LL
T4FH	Taylor	Taylor-4	Pooled	Female	3X	Asexual	Head	LL
T4MB	Taylor	Taylor-4	Pooled	Male	3X	Asexual	Body	LL
T4MH	Taylor	Taylor-4	Pooled	Male	3X	Asexual	Head	LL
G10FB	Gunn	Gunn-10	Pooled	Female	4X	Asexual	Body	LL
G10FH	Gunn	Gunn-10	Pooled	Female	4X	Asexual	Head	LL
G10MB	Gunn	Gunn-10	Pooled	Male	4X	Asexual	Body	LL
G10MH	Gunn	Gunn-10	Pooled	Male	4X	Asexual	Head	LL
G14FB	Gunn	Gunn-14	Pooled	Female	4X	Asexual	Body	LL
G14FH	Gunn	Gunn-14	Pooled	Female	4X	Asexual	Head	LL
G14MB	Gunn	Gunn-14	Pooled	Male	4X	Asexual	Body	LL
G14MH	Gunn	Gunn-14	Pooled	Male	4X	Asexual	Head	LL
R6FB	Rotoiti	Rotoiti-6	Pooled	Female	4X	Asexual	Body	LL
R6FH	Rotoiti	Rotoiti-6	Pooled	Female	4X	Asexual	Head	LL
R6MB	Rotoiti	Rotoiti-6	Pooled	Male	4X	Asexual	Body	LL
R6MH	Rotoiti	Rotoiti-6	Pooled	Male	4X	Asexual	Head	LL

LL=laboratory lineage Field=field collected
