# Supplementary material for: Distinct Bacterial Microbiomes in Sexual and Asexual Potamopyrgus antipodarum, a New Zealand Freshwater Snail
Source: PLoS One. 2016 Aug 26;11(8):e0161050. doi: 10.1371/journal.pone.0161050 (PMC5001651; doi:10.1371/journal.pone.0161050)
Supplement: S2 Table — (PDF) [file pone.0161050.s004.pdf]

S2 Table. Summary of alpha diversity results and non-parametric t-test with 1000 Monte Carlo permutations.

Alpha Metric	Sample Factor	Group 1	Group 2	Group 1 mean	Group 1 SD	Group 2 mean	Group 2 SD	t-stat	*p* -value
**Chao 1**									
	**Reproductive mode**	asexual	sexual	141.20	30.58	127.63	41.45	1.43	0.16
	**Lake**	Kaniere	Rotoiti	150.61	33.51	125.37	20.20	1.26	1.00
		Poerua	Alexandrina	130.24	30.70	173.99	12.22	-2.50	1.00
		Rotoiti	Ianthe	125.37	20.20	104.56	32.78	1.15	1.00
		Gunn	Ianthe	159.45	29.57	104.56	32.78	3.82	0.03
		Rotoiti	Poerua	125.37	20.20	130.24	30.70	-0.26	1.00
		Kaniere	Poerua	150.61	33.51	130.24	30.70	1.19	1.00
		Kaniere	Sarah	150.61	33.51	148.05	28.48	0.15	1.00
		Sarah	Rotoiti	148.05	28.48	125.37	20.20	1.30	1.00
		Rotoiti	Gunn	125.37	20.20	159.45	29.57	-1.90	1.00
		Kaniere	Ianthe	150.61	33.51	104.56	32.78	3.08	0.20
		Sarah	Taylor	148.05	28.48	128.79	20.33	1.10	1.00
		Rotoiti	Alexandrina	125.37	20.20	173.99	12.22	-3.57	1.00
		Taylor	Alexandrina	128.79	20.33	173.99	12.22	-3.30	1.00
		Sarah	Poerua	148.05	28.48	130.24	30.70	1.13	1.00
		Sarah	Gunn	148.05	28.48	159.45	29.57	-0.74	1.00
		Taylor	Poerua	128.79	20.33	130.24	30.70	-0.08	1.00
		Kaniere	Alexandrina	150.61	33.51	173.99	12.22	-1.23	1.00
		Kaniere	Gunn	150.61	33.51	159.45	29.57	-0.52	1.00
		Kaniere	Taylor	150.61	33.51	128.79	20.33	1.09	1.00
		Alexandrina	Gunn	173.99	12.22	159.45	29.57	0.86	1.00
		Poerua	Gunn	130.24	30.70	159.45	29.57	-1.81	1.00
		Sarah	Ianthe	148.05	28.48	104.56	32.78	3.06	0.17
		Taylor	Gunn	128.79	20.33	159.45	29.57	-1.70	1.00
		Sarah	Alexandrina	148.05	28.48	173.99	12.22	-1.59	1.00
		Taylor	Rotoiti	128.79	20.33	125.37	20.20	0.21	1.00
		Taylor	Ianthe	128.79	20.33	104.56	32.78	1.34	1.00
		Poerua	Ianthe	130.24	30.70	104.56	32.78	1.77	1.00
		Alexandrina	Ianthe	173.99	12.22	104.56	32.78	3.95	0.03
	**Lake source**	Field	LL	142.75	29.66	132.90	38.00	0.82	0.43
	**Sex**	Juvenile	Female	122.97	12.18	141.38	40.26	-0.88	1.00
		Male	Female	129.19	33.34	141.38	40.26	-1.20	0.68
		Juvenile	Male	122.97	12.18	129.19	33.34	-0.36	1.00
	**Body section**	Body	Adult	139.45	33.94	173.13	14.71	-1.89	0.36
		Body	Juvenile	139.45	33.94	122.97	12.18	0.93	1.00
		Juvenile	Adult	122.97	12.18	173.13	14.71	-4.55	0.13
		Body	Head	139.45	33.94	126.24	39.46	1.27	1.00
		Juvenile	Head	122.97	12.18	126.24	39.46	-0.16	1.00
		Head	Adult	126.24	39.46	173.13	14.71	-2.27	0.17
	**Ploidy level**	3X	2X	137.07	29.41	127.63	41.45	0.86	1.00
		2X	4X	127.63	41.45	148.09	31.26	-1.49	0.44
		3X	4X	137.07	29.41	148.09	31.26	-0.97	1.00
**Dominance**									
	**Reproductive mode**	asexual	sexual	0.09	0.05	0.16	0.14	-2.66	0.01
	**Lake**	Kaniere	Rotoiti	0.10	0.08	0.08	0.03	0.42	1.00
		Poerua	Alexandrina	0.11	0.10	0.04	0.00	1.28	1.00
		Rotoiti	Ianthe	0.08	0.03	0.21	0.14	-1.81	1.00
		Gunn	Ianthe	0.06	0.02	0.21	0.14	-2.81	0.39
		Rotoiti	Poerua	0.08	0.03	0.11	0.10	-0.63	1.00
		Kaniere	Poerua	0.10	0.08	0.11	0.10	-0.30	1.00
		Kaniere	Sarah	0.10	0.08	0.09	0.02	0.14	1.00
		Sarah	Rotoiti	0.09	0.02	0.08	0.03	0.99	1.00
		Rotoiti	Gunn	0.08	0.03	0.06	0.02	0.77	1.00
		Kaniere	Ianthe	0.10	0.08	0.21	0.14	-2.06	1.00
		Sarah	Taylor	0.09	0.02	0.07	0.02	1.82	1.00
		Rotoiti	Alexandrina	0.08	0.03	0.04	0.00	2.03	1.00
		Taylor	Alexandrina	0.07	0.02	0.04	0.00	2.88	0.48
		Sarah	Poerua	0.09	0.02	0.11	0.10	-0.50	1.00
		Sarah	Gunn	0.09	0.02	0.06	0.02	2.62	0.62
		Taylor	Poerua	0.07	0.02	0.11	0.10	-0.77	1.00
		Kaniere	Alexandrina	0.10	0.08	0.04	0.00	1.18	1.00
		Kaniere	Gunn	0.10	0.08	0.06	0.02	0.98	1.00
		Kaniere	Taylor	0.10	0.08	0.07	0.02	0.59	1.00
		Alexandrina	Gunn	0.04	0.00	0.06	0.02	-2.11	1.00
		Poerua	Gunn	0.11	0.10	0.06	0.02	1.23	1.00
		Sarah	Ianthe	0.09	0.02	0.21	0.14	-2.30	0.87
		Taylor	Gunn	0.07	0.02	0.06	0.02	0.38	1.00
		Sarah	Alexandrina	0.09	0.02	0.04	0.00	4.57	0.14
		Taylor	Rotoiti	0.07	0.02	0.08	0.03	-0.37	1.00
		Taylor	Ianthe	0.07	0.02	0.21	0.14	-1.91	1.00
		Poerua	Ianthe	0.11	0.10	0.21	0.14	-1.76	1.00
		Alexandrina	Ianthe	0.04	0.00	0.21	0.14	-2.27	1.00
	**Lake source**	Field	LL	0.11	0.06	0.12	0.12	-0.34	0.73

	**Sex**	Juvenile	Female	0.10	0.02	0.12	0.11	-0.19	1.00
		Male	Female	0.12	0.11	0.12	0.11	0.25	1.00
		Juvenile	Male	0.10	0.02	0.12	0.11	-0.34	1.00
	**Body section**	Body	Adult	0.10	0.09	0.08	0.01	0.40	1.00
		Body	Juvenile	0.10	0.09	0.10	0.02	-0.14	1.00
		Juvenile	Adult	0.10	0.02	0.08	0.01	2.05	0.50
		Body	Head	0.10	0.09	0.15	0.13	-1.52	0.81
		Juvenile	Head	0.10	0.02	0.15	0.13	-0.62	1.00
		Head	Adult	0.15	0.13	0.08	0.01	0.99	1.00
	**Ploidy level**	3X	2X	0.10	0.06	0.16	0.14	-1.82	0.23
		2X	4X	0.16	0.14	0.07	0.02	2.15	0.11
		3X	4X	0.10	0.06	0.07	0.02	1.31	0.58
**Equitability**									
	**Reproductive mode**	asexual	sexual	0.76	0.07	0.71	0.14	2.05	0.04
	**Lake**	Kaniere	Rotoiti	0.77	0.10	0.77	0.06	0.01	1.00
		Poerua	Alexandrina	0.75	0.10	0.83	0.01	-1.47	1.00
		Rotoiti	Ianthe	0.77	0.06	0.65	0.13	1.78	1.00
		Gunn	Ianthe	0.80	0.03	0.65	0.13	3.11	0.17
		Rotoiti	Poerua	0.77	0.06	0.75	0.10	0.37	1.00
		Kaniere	Poerua	0.77	0.10	0.75	0.10	0.42	1.00
		Kaniere	Sarah	0.77	0.10	0.73	0.04	0.92	1.00
		Sarah	Rotoiti	0.73	0.04	0.77	0.06	-1.27	1.00
		Rotoiti	Gunn	0.77	0.06	0.80	0.03	-1.00	1.00
		Kaniere	Ianthe	0.77	0.10	0.65	0.13	2.27	0.98
		Sarah	Taylor	0.73	0.04	0.79	0.02	-2.50	1.00
		Rotoiti	Alexandrina	0.77	0.06	0.83	0.01	-1.84	1.00
		Taylor	Alexandrina	0.79	0.02	0.83	0.01	-3.02	1.00
		Sarah	Poerua	0.73	0.04	0.75	0.10	-0.38	1.00
		Sarah	Gunn	0.73	0.04	0.80	0.03	-3.66	0.11
		Taylor	Poerua	0.79	0.02	0.75	0.10	0.72	1.00
		Kaniere	Alexandrina	0.77	0.10	0.83	0.01	-1.05	1.00
		Kaniere	Gunn	0.77	0.10	0.80	0.03	-0.64	1.00
		Kaniere	Taylor	0.77	0.10	0.79	0.02	-0.31	1.00
		Alexandrina	Gunn	0.83	0.01	0.80	0.03	2.25	1.00
		Poerua	Gunn	0.75	0.10	0.80	0.03	-1.22	1.00
		Sarah	Ianthe	0.73	0.04	0.65	0.13	1.76	1.00
		Taylor	Gunn	0.79	0.02	0.80	0.03	-0.46	1.00
		Sarah	Alexandrina	0.73	0.04	0.83	0.01	-4.68	0.06
		Taylor	Rotoiti	0.79	0.02	0.77	0.06	0.53	1.00
		Taylor	Ianthe	0.79	0.02	0.65	0.13	2.08	1.00
		Poerua	Ianthe	0.75	0.10	0.65	0.13	1.85	1.00
		Alexandrina	Ianthe	0.83	0.01	0.65	0.13	2.69	0.36
	**Lake source**	Field	LL	0.72	0.07	0.74	0.12	-0.47	0.64
	**Sex**	Juvenile	Female	0.70	0.03	0.75	0.11	-0.80	1.00
		Male	Female	0.73	0.11	0.75	0.11	-0.51	1.00
		Juvenile	Male	0.70	0.03	0.73	0.11	-0.54	1.00
	**Body section**	Body	Adult	0.76	0.10	0.76	0.01	-0.01	1.00
		Body	Juvenile	0.76	0.10	0.70	0.03	1.18	1.00
		Juvenile	Adult	0.70	0.03	0.76	0.01	-3.39	0.11
		Body	Head	0.76	0.10	0.71	0.12	1.52	0.84
		Juvenile	Head	0.70	0.03	0.71	0.12	-0.21	1.00
		Head	Adult	0.71	0.12	0.76	0.01	-0.76	1.00
	**Ploidy level**	3X	2X	0.75	0.07	0.71	0.14	1.24	0.66
		2X	4X	0.71	0.14	0.79	0.04	-1.96	0.15
		3X	4X	0.75	0.07	0.79	0.04	-1.61	0.35
**Faith's Phylogenetic Diversity**									
	**Reproductive mode**	asexual	sexual	9.50	1.33	8.87	1.94	1.46	0.18
	**Lake**	Kaniere	Rotoiti	9.92	2.28	8.79	0.94	0.87	1.00
		Poerua	Alexandrina	8.66	1.53	9.61	0.64	-1.09	1.00
		Rotoiti	Ianthe	8.79	0.94	8.16	1.64	0.70	1.00
		Gunn	Ianthe	10.47	0.96	8.16	1.64	3.54	0.14
		Rotoiti	Poerua	8.79	0.94	8.66	1.53	0.14	1.00
		Kaniere	Poerua	9.92	2.28	8.66	1.53	1.22	1.00
		Kaniere	Sarah	9.92	2.28	10.02	0.91	-0.10	1.00
		Sarah	Rotoiti	10.02	0.91	8.79	0.94	2.00	1.00
		Rotoiti	Gunn	8.79	0.94	10.47	0.96	-2.65	0.48
		Kaniere	Ianthe	9.92	2.28	8.16	1.64	2.08	1.00
		Sarah	Taylor	10.02	0.91	8.91	0.62	2.01	1.00
		Rotoiti	Alexandrina	8.79	0.94	9.61	0.64	-1.26	1.00
		Taylor	Alexandrina	8.91	0.62	9.61	0.64	-1.37	1.00
		Sarah	Poerua	10.02	0.91	8.66	1.53	2.02	1.00
		Sarah	Gunn	10.02	0.91	10.47	0.96	-0.92	1.00
		Taylor	Poerua	8.91	0.62	8.66	1.53	0.29	1.00
		Kaniere	Alexandrina	9.92	2.28	9.61	0.64	0.24	1.00

		Kaniere	Gunn	9.92	2.28	10.47	0.96	-0.59	1.00
		Kaniere	Taylor	9.92	2.28	8.91	0.62	0.80	1.00
		Alexandrina	Gunn	9.61	0.64	10.47	0.96	-1.49	1.00
		Poerua	Gunn	8.66	1.53	10.47	0.96	-2.67	0.76
		Sarah	Ianthe	10.02	0.91	8.16	1.64	2.86	0.34
		Taylor	Gunn	8.91	0.62	10.47	0.96	-2.72	0.64
		Sarah	Alexandrina	10.02	0.91	9.61	0.64	0.73	1.00
		Taylor	Rotoiti	8.91	0.62	8.79	0.94	0.19	1.00
		Taylor	Ianthe	8.91	0.62	8.16	1.64	0.85	1.00
		Poerua	Ianthe	8.66	1.53	8.16	1.64	0.69	1.00
		Alexandrina	Ianthe	9.61	0.64	8.16	1.64	1.65	1.00
	**Lake source**	Field	LL	9.40	1.50	9.16	1.71	0.45	0.66
	**Sex**	Juvenile	Female	9.52	0.79	9.36	1.75	0.18	1.00
		Male	Female	8.99	1.65	9.36	1.75	-0.80	1.00
		Juvenile	Male	9.52	0.79	8.99	1.65	0.62	1.00
	**Body section**	Body	Adult	9.44	1.72	10.51	0.72	-1.19	1.00
		Body	Juvenile	9.44	1.72	9.52	0.79	-0.08	1.00
		Juvenile	Adult	9.52	0.79	10.51	0.72	-1.61	0.94
		Body	Head	9.44	1.72	8.72	1.66	1.52	0.89
		Juvenile	Head	9.52	0.79	8.72	1.66	0.92	1.00
		Head	Adult	8.72	1.66	10.51	0.72	-2.06	0.32
	**Ploidy level**	3X	2X	9.25	1.32	8.87	1.94	0.75	1.00
		2X	4X	8.87	1.94	9.91	1.24	-1.67	0.29
		3X	4X	9.25	1.32	9.91	1.24	-1.36	0.49
**Shannon**									
	**Reproductive mode**	asexual	sexual	4.87	0.56	4.42	1.14	1.93	0.06
	**Lake**	Kaniere	Rotoiti	4.98	0.94	4.75	0.45	0.41	1.00
		Poerua	Alexandrina	4.74	0.85	5.50	0.03	-1.64	1.00
		Rotoiti	Ianthe	4.75	0.45	3.87	1.05	1.56	1.00
		Gunn	Ianthe	5.20	0.27	3.87	1.05	3.37	0.22
		Rotoiti	Poerua	4.75	0.45	4.74	0.85	0.03	1.00
		Kaniere	Poerua	4.98	0.94	4.74	0.85	0.50	1.00
		Kaniere	Sarah	4.98	0.94	4.68	0.39	0.79	1.00
		Sarah	Rotoiti	4.68	0.39	4.75	0.45	-0.28	1.00
		Rotoiti	Gunn	4.75	0.45	5.20	0.27	-1.94	1.00
		Kaniere	Ianthe	4.98	0.94	3.87	1.05	2.41	0.62
		Sarah	Taylor	4.68	0.39	4.99	0.20	-1.38	1.00
		Rotoiti	Alexandrina	4.75	0.45	5.50	0.03	-2.86	0.67
		Taylor	Alexandrina	4.99	0.20	5.50	0.03	-4.45	0.48
		Sarah	Poerua	4.68	0.39	4.74	0.85	-0.17	1.00
		Sarah	Gunn	4.68	0.39	5.20	0.27	-2.92	0.34
		Taylor	Poerua	4.99	0.20	4.74	0.85	0.54	1.00
		Kaniere	Alexandrina	4.98	0.94	5.50	0.03	-1.03	1.00
		Kaniere	Gunn	4.98	0.94	5.20	0.27	-0.60	1.00
		Kaniere	Taylor	4.98	0.94	4.99	0.20	-0.02	1.00
		Alexandrina	Gunn	5.50	0.03	5.20	0.27	2.08	1.00
		Poerua	Gunn	4.74	0.85	5.20	0.27	-1.37	1.00
		Sarah	Ianthe	4.68	0.39	3.87	1.05	2.00	1.00
		Taylor	Gunn	4.99	0.20	5.20	0.27	-1.26	1.00
		Sarah	Alexandrina	4.68	0.39	5.50	0.03	-3.87	0.14
		Taylor	Rotoiti	4.99	0.20	4.75	0.45	0.83	1.00
		Taylor	Ianthe	4.99	0.20	3.87	1.05	2.01	1.00
		Poerua	Ianthe	4.74	0.85	3.87	1.05	1.93	1.00
		Alexandrina	Ianthe	5.50	0.03	3.87	1.05	2.95	0.25
	**Lake source**	Field	LL	4.59	0.64	4.68	0.96	-0.31	0.76
	**Sex**	Juvenile	Female	4.35	0.27	4.76	0.96	-0.82	1.00
		Male	Female	4.60	0.90	4.76	0.96	-0.64	1.00
		Juvenile	Male	4.35	0.27	4.60	0.90	-0.52	1.00
	**Body section**	Body	Adult	4.87	0.87	5.00	0.15	-0.29	1.00
		Body	Juvenile	4.87	0.87	4.35	0.27	1.13	1.00
		Juvenile	Adult	4.35	0.27	5.00	0.15	-3.58	0.05
		Body	Head	4.87	0.87	4.45	1.01	1.55	0.86
		Juvenile	Head	4.35	0.27	4.45	1.01	-0.19	1.00
		Head	Adult	4.45	1.01	5.00	0.15	-1.05	1.00
	**Ploidy level**	3X	2X	4.76	0.61	4.42	1.14	1.19	0.76
		2X	4X	4.42	1.14	5.05	0.40	-1.81	0.21
		3X	4X	4.76	0.61	5.05	0.40	-1.40	0.49
**Good's Coverage**									
	**Reproductive mode**	asexual	sexual	0.90	0.02	0.91	0.03	-1.76	0.10
	**Lake**	Kaniere	Rotoiti	0.89	0.03	0.91	0.01	-1.40	1.00
		Poerua	Alexandrina	0.91	0.02	0.88	0.00	2.17	1.00
		Rotoiti	Ianthe	0.91	0.01	0.93	0.02	-0.96	1.00
		Gunn	Ianthe	0.89	0.02	0.93	0.02	-3.87	0.06

	Rotoiti	Poerua	0.91	0.01	0.91	0.02	0.67	1.00
	Kaniere	Poerua	0.89	0.03	0.91	0.02	-0.91	1.00
	Kaniere	Sarah	0.89	0.03	0.89	0.02	0.41	1.00
	Sarah	Rotoiti	0.89	0.02	0.91	0.01	-2.39	0.90
	Rotoiti	Gunn	0.91	0.01	0.89	0.02	2.08	1.00
	Kaniere	Ianthe	0.89	0.03	0.93	0.02	-3.01	0.22
	Sarah	Taylor	0.89	0.02	0.91	0.02	-1.98	1.00
	Rotoiti	Alexandrina	0.91	0.01	0.88	0.00	5.08	0.39
	Taylor	Alexandrina	0.91	0.02	0.88	0.00	3.57	0.36
	Sarah	Poerua	0.89	0.02	0.91	0.02	-1.53	1.00
	Sarah	Gunn	0.89	0.02	0.89	0.02	0.32	1.00
	Taylor	Poerua	0.91	0.02	0.91	0.02	0.43	1.00
	Kaniere	Alexandrina	0.89	0.03	0.88	0.00	1.09	1.00
	Kaniere	Gunn	0.89	0.03	0.89	0.02	0.64	1.00
	Kaniere	Taylor	0.89	0.03	0.91	0.02	-1.16	1.00
	Alexandrina	Gunn	0.88	0.00	0.89	0.02	-0.53	1.00
	Poerua	Gunn	0.91	0.02	0.89	0.02	1.61	1.00
	Sarah	Ianthe	0.89	0.02	0.93	0.02	-3.87	0.06
	Taylor	Gunn	0.91	0.02	0.89	0.02	1.80	1.00
	Sarah	Alexandrina	0.89	0.02	0.88	0.00	1.09	1.00
	Taylor	Rotoiti	0.91	0.02	0.91	0.01	-0.25	1.00
	Taylor	Ianthe	0.91	0.02	0.93	0.02	-1.16	1.00
	Poerua	Ianthe	0.91	0.02	0.93	0.02	-2.01	1.00
	Alexandrina	Ianthe	0.88	0.00	0.93	0.02	-3.92	0.06
**Lake source**	Field	LL	0.90	0.02	0.91	0.03	-1.26	0.23
**Sex**	Juvenile	Female	0.90	0.01	0.90	0.03	0.22	1.00
	Male	Female	0.91	0.02	0.90	0.03	0.91	1.00
	Juvenile	Male	0.90	0.01	0.91	0.02	-0.27	1.00
**Body section**	Body	Adult	0.90	0.02	0.87	0.01	2.17	0.19
	Body	Juvenile	0.90	0.02	0.90	0.01	-0.21	1.00
	Juvenile	Adult	0.90	0.01	0.87	0.01	4.30	0.07
	Body	Head	0.90	0.02	0.91	0.03	-1.23	1.00
	Juvenile	Head	0.90	0.01	0.91	0.03	-0.44	1.00
	Head	Adult	0.91	0.03	0.87	0.01	2.43	0.14
**Ploidy level**	3X	2X	0.90	0.02	0.91	0.03	-1.29	0.57
	2X	4X	0.91	0.03	0.90	0.02	1.53	0.37
	3X	4X	0.90	0.02	0.90	0.02	0.58	1.00

LL=laboratory lineage Field=field collected
