# Supplementary material for: Distinct Bacterial Microbiomes in Sexual and Asexual Potamopyrgus antipodarum, a New Zealand Freshwater Snail
Source: PLoS One. 2016 Aug 26;11(8):e0161050. doi: 10.1371/journal.pone.0161050 (PMC5001651; doi:10.1371/journal.pone.0161050)
Supplement: S3 Table — (PDF) [file pone.0161050.s005.pdf]

S3 Table. Analysis of similarity (ANOSIM) of Bray-Curtis distances among snail bacterial communities by reproductive mode.

Sample Factor	Significant Pairwise Comparisons		R-statistic (overall)	R-statistic (pairwise)	*p* value[a]
**Reproductive mode**	**Sexual**	**Asexual**	0.274		0.001
		3x *vs.* 4x		0.114	0.056
**By sex**	Males	Males		0.258	0.002
	Females	Males		0.264	0.002
	Males	Females		0.269	0.001
	Females	Females		0.325	0.001
**By body section**	Head	Body		0.340	0.001
	Head	Head		0.345	0.001
	Body	Body		0.404	0.001
	Body	Head		0.497	0.001
	Head:Body			0.111	0.03

[a]*p* values less than 0.05 were considered statistically significant. While the Global R-statitic of the Bray-Curtis distance for Ploidy was not significant and only marginally significant for 3x vs. 4x, we include it here because ANOSIM results for Unifrac distances were significant (data not shown) and random forests analysis strongly supported the significance of this factor.
