# Supplementary material for: Distinct Bacterial Microbiomes in Sexual and Asexual Potamopyrgus antipodarum, a New Zealand Freshwater Snail
Source: PLoS One. 2016 Aug 26;11(8):e0161050. doi: 10.1371/journal.pone.0161050 (PMC5001651; doi:10.1371/journal.pone.0161050)
Supplement: S4 Table — (PDF) [file pone.0161050.s006.pdf]

S4 Table. Analysis of similarity (ANOSIM) of Bray-Curtis distances among snail bacterial communities by snail population.

Sample Factor	Significant Pairwise Comparisons		R-statistic (pairwise)	*p* value
**Population**				0.001
	Peorua-3	Taylor-4	0.115	0.157
	Peorua-3	Peorua-4	0.135	0.157
	Peorua-4	Taylor-4	0.448	0.043
	Kaniere-1	Peorua-3	0.458	0.043
	Ianthe	Sarah-Juveniles	0.508	0.003
	Peorua-3	Rotoiti-6	0.531	0.014
	Kaniere-1	Sarah-Adult	0.594	0.014
	Kaniere-1	Peorua-4	0.635	0.014
	Kaniere-1	Taylor-4	0.646	0.014
	Kaniere-1	Ianthe	0.655	0.001
	Gunn-10	Peorua-3	0.677	0.014
	Peorua-3	Gunn-14	0.688	0.014
	Sarah-Adult	Ianthe	0.690	0.001
	Rotoiti-6	Ianthe	0.694	0.002
	Gunn-10	Ianthe	0.725	0.001
	Gunn-10	Kaniere	0.781	0.014
	Ianthe	Kaniere	0.786	0.001
	Peorua-3	Kaniere	0.802	0.014
	Ianthe	Gunn-14	0.809	0.001
	Gunn-10	Rotoiti-6	0.813	0.014
	Rotoiti-6	Taylor-4	0.813	0.014
	Peorua-3	Ianthe	0.834	0.001
	Rotoiti-6	Gunn-14	0.844	0.014
	Peorua-3	Sarah-Adult	0.865	0.014
	Ianthe	Peorua-4	0.870	0.001
	Alexandrina	Peorua-3	0.875	0.014
	Alexandrina	Ianthe	0.882	0.001
	Ianthe	Taylor-4	0.891	0.001
	Kaniere-1	Rotoiti-6	0.896	0.014
	Peorua-3	Sarah-Juveniles	0.896	0.014
	Gunn-14	Peorua-4	0.906	0.014
	Alexandrina	Kaniere-1	0.917	0.014
	Gunn-10	Taylor-4	0.917	0.014
	Sarah-Adult	Sarah-Juveniles	0.917	0.014
	Rotoiti-6	Sarah-Juveniles	0.938	0.014
	Alexandrina	Gunn-14	0.948	0.014
	Gunn-10	Peorua-4	0.948	0.014
	Gunn-10	Kaniere-1	0.958	0.014
	Rotoiti-6	Kaniere	0.958	0.014
	Rotoiti-6	Peorua-4	0.958	0.014
	Gunn-14	Kaniere	0.958	0.014
	Kaniere	Peorua-4	0.969	0.014

Kaniere-1	Gunn-14	0.979	0.014
Kaniere-1	Kaniere	0.990	0.014
Sarah-Adult	Peorua-4	0.990	0.014
Alexandrina	Gunn-10	1.000	0.014
Alexandrina	Rotoiti-6	1.000	0.014
Alexandrina	Sarah-Adult	1.000	0.014
Alexandrina	Kaniere	1.000	0.014
Alexandrina	Peorua-4	1.000	0.014
Alexandrina	Sarah-Juveniles	1.000	0.014
Alexandrina	Taylor-4	1.000	0.014
Gunn-10	Sarah-Adult	1.000	0.014
Gunn-10	Gunn-14	1.000	0.014
Gunn-10	Sarah-Juveniles	1.000	0.014
Kaniere-1	Sarah-Juveniles	1.000	0.014
Rotoiti-6	Sarah-Adult	1.000	0.014
Sarah-Adult	Gunn-14	1.000	0.014
Sarah-Adult	Kaniere	1.000	0.014
Sarah-Adult	Taylor-4	1.000	0.014
Gunn-14	Sarah-Juveniles	1.000	0.014
Gunn-14	Taylor-4	1.000	0.014
Kaniere	Sarah-Juveniles	1.000	0.014
Kaniere	Taylor-4	1.000	0.014
Peorua-4	Sarah-Juveniles	1.000	0.014
Sarah-Juveniles	Taylor-4	1.000	0.014

All Ianthe and Sarah samples were pooled in this comparison because each of these samples represented an individual snail whereas the other replicates contained DNA from 3 pooled snails.
