# Supplementary material for: Distinct Bacterial Microbiomes in Sexual and Asexual Potamopyrgus antipodarum, a New Zealand Freshwater Snail
Source: PLoS One. 2016 Aug 26;11(8):e0161050. doi: 10.1371/journal.pone.0161050 (PMC5001651; doi:10.1371/journal.pone.0161050)
Supplement: S5 Table — (PDF) [file pone.0161050.s007.pdf]

S5 Table. Estimates of 16S rRNA gene divergence between OTU sequences identified as significant in SIMPER and nearest BLAST

OTU or Nearest BLAST match	2	3	4	5	6	7	8	9	10	11	12	13	14	15	16	17	18	19	20	21	22	23	24	25	26	27	28	29	30	31	32	33	34	35	36
1. OTU1014	0.191	0.191	0.222	0.204	0.308	0.217	0.222	0.191	0.231	0.195	0.182	0.128	0.235	0.182	0.222	0.124	0.191	0.182	0.186	0.254	0.182	0.204	0.222	0.120	0.148	0.217	0.240	0.195	0.208	0.186	0.195	0.000	0.153	0.231	0.169
2. OTU104		0.213	0.204	0.161	0.339	0.195	0.231	0.165	0.278	0.165	0.195	0.208	0.191	0.165	0.182	0.204	0.195	0.199	0.182	0.249	0.178	0.161	0.191	0.208	0.136	0.169	0.235	0.191	0.165	0.191	0.217	0.191	0.157	0.169	0.245
3. OTU1327			0.148	0.140	0.308	0.217	0.293	0.217	0.254	0.132	0.128	0.208	0.153	0.148	0.226	0.213	0.014	0.235	0.128	0.235	0.116	0.226	0.144	0.208	0.222	0.222	0.240	0.101	0.144	0.148	0.031	0.191	0.213	0.165	0.226
4. OTU1691				0.132	0.339	0.191	0.283	0.186	0.288	0.108	0.157	0.208	0.056	0.128	0.204	0.213	0.148	0.226	0.157	0.222	0.153	0.191	0.049	0.208	0.199	0.191	0.213	0.136	0.128	0.116	0.153	0.222	0.208	0.153	0.259
5. OTU1813					0.350	0.169	0.254	0.148	0.254	0.112	0.112	0.165	0.093	0.178	0.165	0.169	0.140	0.173	0.108	0.222	0.105	0.153	0.097	0.169	0.173	0.169	0.213	0.086	0.017	0.128	0.144	0.204	0.173	0.191	0.249
6. OTU182						0.334	0.350	0.318	0.254	0.293	0.328	0.308	0.344	0.298	0.344	0.303	0.288	0.328	0.344	0.148	0.339	0.323	0.350	0.298	0.303	0.334	0.161	0.344	0.355	0.288	0.303	0.308	0.360	0.350	0.245
7. OTU228							0.199	0.027	0.231	0.182	0.186	0.208	0.178	0.173	0.031	0.213	0.217	0.124	0.208	0.226	0.213	0.042	0.169	0.199	0.199	0.027	0.217	0.213	0.173	0.173	0.208	0.217	0.116	0.182	0.235
8. OTU2405								0.191	0.273	0.226	0.259	0.217	0.264	0.195	0.213	0.217	0.283	0.153	0.264	0.308	0.268	0.195	0.273	0.217	0.217	0.204	0.308	0.249	0.249	0.191	0.298	0.222	0.144	0.213	0.245
9. OTU270									0.222	0.169	0.199	0.191	0.173	0.195	0.034	0.186	0.199	0.108	0.204	0.213	0.208	0.017	0.165	0.182	0.178	0.031	0.199	0.199	0.153	0.169	0.213	0.191	0.097	0.199	0.226
10. OTU362										0.273	0.254	0.283	0.273	0.259	0.235	0.278	0.235	0.208	0.259	0.173	0.268	0.235	0.278	0.283	0.288	0.235	0.186	0.288	0.259	0.240	0.240	0.231	0.222	0.293	0.116
11. OTU549											0.116	0.161	0.112	0.101	0.204	0.157	0.116	0.195	0.108	0.204	0.101	0.182	0.108	0.169	0.178	0.191	0.182	0.063	0.116	0.089	0.136	0.195	0.169	0.144	0.217
12. OTU608												0.182	0.173	0.116	0.208	0.191	0.128	0.199	0.020	0.226	0.034	0.208	0.153	0.182	0.199	0.213	0.217	0.105	0.116	0.097	0.140	0.182	0.195	0.128	0.235
13. OTU821													0.199	0.217	0.213	0.010	0.208	0.191	0.191	0.240	0.186	0.208	0.199	0.007	0.153	0.208	0.235	0.169	0.161	0.195	0.204	0.128	0.157	0.245	0.226
14. OTU969														0.128	0.191	0.204	0.153	0.222	0.165	0.208	0.161	0.178	0.024	0.208	0.178	0.178	0.213	0.101	0.105	0.136	0.153	0.235	0.222	0.148	0.240
15. OTU985															0.195	0.222	0.148	0.165	0.124	0.231	0.120	0.195	0.128	0.217	0.191	0.173	0.208	0.132	0.182	0.093	0.144	0.182	0.173	0.049	0.199
16. *Leptothrix ginsengisoli* str. OTSz A_210 (FM886840.1)																0.217	0.226	0.093	0.226	0.245	0.231	0.031	0.182	0.204	0.204	0.024	0.235	0.226	0.169	0.195	0.222	0.222	0.112	0.204	0.245
17. *Rheinheimera* sp. str. 09BSZB-9 (HM566014.1)																	0.213	0.191	0.191	0.235	0.182	0.204	0.204	0.010	0.165	0.213	0.231	0.173	0.165	0.204	0.208	0.124	0.153	0.249	0.226
18. *Phenylobacterium* sp. str. A8 (HM047736.1)																		0.217	0.128	0.217	0.116	0.208	0.144	0.208	0.204	0.222	0.222	0.101	0.144	0.132	0.045	0.191	0.204	0.161	0.208
19. *Paucimonas lemoignei* str. LMG 2207 (NR_026276.1)																			0.213	0.259	0.217	0.105	0.222	0.182	0.213	0.112	0.254	0.213	0.178	0.144	0.222	0.182	0.078	0.195	0.208
20. *Sphingomonas* sp. BAC151 (EU131005.1)																				0.231	0.014	0.208	0.153	0.199	0.199	0.231	0.222	0.097	0.112	0.108	0.140	0.186	0.195	0.136	0.240
21. *Fervidomicrobium thiophilum* str. SR (EF554597.1)																					0.231	0.231	0.204	0.240	0.245	0.231	0.060	0.240	0.226	0.204	0.231	0.254	0.254	0.254	0.191
22. *Sphingomonas* sp. str. BCB-54 (AB288319.1)																						0.213	0.148	0.195	0.204	0.235	0.217	0.093	0.108	0.105	0.128	0.182	0.199	0.132	0.245
23. *Methylibium petroleiphilum* str. PM1 (CP000555.1)																							0.173	0.199	0.186	0.027	0.222	0.208	0.157	0.178	0.213	0.204	0.101	0.199	0.231
24. *Rhodobacter* sp. str. TUT3731 (AB251407.1)																								0.199	0.195	0.169	0.208	0.101	0.108	0.116	0.148	0.222	0.213	0.144	0.254
25. *Rheinheimera* sp. str. J3-AN42 (DQ454129.1)																									0.161	0.199	0.235	0.178	0.165	0.195	0.204	0.120	0.148	0.245	0.226
26. *Anaplasma centrale* (AB211164.1)																										0.178	0.231	0.191	0.169	0.186	0.222	0.148	0.195	0.178	0.208
27. *Rubrivivax indolicus* str. OU5 (AJ620346.1)																											0.222	0.222	0.173	0.182	0.208	0.217	0.108	0.182	0.240
28. *Carboxydothermus hydrogenoformans* str. Z-2901 (CP000141.1)																												0.226	0.217	0.182	0.235	0.240	0.254	0.245	0.178
29. *Sinorhizobium* sp. str. R-24605 (AM084000.1)																													0.097	0.097	0.105	0.195	0.182	0.140	0.235
30. *Hyphomicrobium facile* (AB222020.1)																														0.132	0.148	0.208	0.178	0.186	0.245
31. *Methylobacterium* sp. str. PB138 (AB220085.1)																															0.144	0.186	0.165	0.112	0.199
32. *Phenylobacterium lituiforme* str. FaiI3 (AY534887.1)																																0.195	0.191	0.161	0.222
33. *Pseudomonas* sp. str. 12A_19 (AY689078.1)																																	0.153	0.231	0.169
34. *Derxia gummosa* str. IAM13946 (AB089482.1)																																		0.191	0.222
35. *Candidatus* Odyssella thessalonicensis str. L13 (AF069496.1)																																			0.240
36. *Holophaga foetida* (X77215.1)																																			

The dissimilairty between DNA sequences are shown. Analyses were conducted using the Jukes-Cantor model and the analysis involved 36 nucleotide sequences. All positions containing gaps and missing data were eliminated. There were a total of 297 positions in the final dataset.
